# High-flow versus low-flow nasal oxygen during procedural sedation for atrial fibrillation ablation: A randomised controlled trial

**DOI:** 10.1097/EA9.0000000000000129

**Published:** 2026-07-29

**Authors:** Marloes Homberg, Dominik Linz, Lloyd Brandts, E.A. Joosten, Wolfgang Buhre, Esther Bouman

**Affiliations:** From the Department of Anaesthesiology, Maastricht University Medical Centre, Maastricht, The Netherlands (MH, EAJ, EB), Department of Cardiology, Maastricht University Medical Centre and Cardiovascular Research Institute Maastricht, Maastricht, The Netherlands (DL), Department of Biomedical Sciences, Faculty of Health and Medical Sciences, University of Copenhagen, Copenhagen, Denmark (DL), Department of Clinical Epidemiology and Medical Technology Assessment (KEMTA), Maastricht University Medical Centre, Maastricht, The Netherlands (LB), Department of Anaesthesiology, University Medical Centre Utrecht, Utrecht, The Netherlands (WB)

Editor,

Procedural sedation and analgesia (PSA) for atrial fibrillation (AF) catheter ablation is associated with a risk of oxygen desaturation, particularly during prolonged deep sedation.^1^ At our institution, PSA is routinely used for AF catheter ablation, with general anaesthesia reserved for patients with a contraindication to PSA. High-flow nasal oxygen (HFNO) may improve oxygenation by delivering heated, humidified gas at high flow rates and generating low positive airway pressure.^2^ Evidence for HFNO during PSA remains inconsistent. This randomised controlled trial (RCT) investigated whether HFNO improves oxygenation compared with low-flow nasal oxygen (LFNO) during PSA for AF catheter ablation.

This single-centre RCT was conducted at Maastricht University Medical Centre+. Adults undergoing elective point-by-point AF catheter ablation during PSA were randomised 1 : 1 to HFNO or LFNO.^3^ Exclusion criteria included BMI >32 kg m^-2^, diagnosed sleep-disordered breathing, severe chronic obstructive pulmonary disease, chronic oxygen dependency, anticipated difficult airway, or a contra-indication for HFNO. Ethical approval (METC20-072) was granted by the Medical Ethics Review Committee Maastricht, the Netherlands, on 23 December 2020. Study registration at ClinicalTrials.gov (identifier: NCT04842253).

HFNO was delivered via Optiflow at 50 l min^−1^; with a fixed oxygen–air blend of F_I_O_2_ 40%. Oral/nasopharyngeal airway adjuncts were not permitted during HFNO therapy to avoid loss of pharyngeal pressure. LFNO was administered at 5 l min^−1^ via nasal cannula.^4^ Oral/nasopharyngeal airways were permitted in the LFNO group if obstruction occurred. Sedation was propofol-based and provided by trained anaesthesia staff under supervision.^3^ Monitoring included bispectral index (BIS), capnography, and transcutaneous CO_2_ (TcCO_2_).^3^

Primary outcomes were lowest SpO_2_ and desaturations duration. Secondary outcomes included hypoxaemia (SpO_2_ <90% for >180 s), area under the desaturation curve (AUCDESAT), crossover of oxygen modality, TcCO_2_ and adverse events. Between-group comparisons were performed using independent-samples Student's *t*-tests according to the intention-to-treat principle. The study was terminated early after inclusion of 101 participants (54% of the intended sample size) due to declining recruitment and feasibility constraints.

Between December 2021 and November 2024, 554 patients were screened, 104 randomised, and 101 analysed (HFNO *n* = 51; LFNO *n* = 50). Baseline characteristics were comparable between groups.

Mean lowest SpO_2_ was 89.6% ± 6.6 in the HFNO group and 87.7% ± 5.9 in the LFNO group (*P* = 0.127). Desaturation duration was longer with HFNO (55.7 ± 99.4 s vs. 41.3 ± 54.2 s; *P* = 0.002).

Crossover occurred in 13.7% of HFNO cases and 6.0% of LFNO cases (*P* = 0.205). Sedation-related complications occurred in 39.2% of HFNO and 48.0% of LFNO cases, mainly airway obstruction, short desaturation, hypotension requiring vasopressors, or airway manoeuvres. One hypoxaemic event occurred in the LFNO group (AUCDESAT 240 s %); none in the HFNO group. Most complications were mild and resolved with standard interventions. The number of declared adverse events (AEs) and serious adverse events (SAEs) reflects only those meeting predefined criteria and does not fully capture transient, nonserious events contributing to the overall complication rate. AEs rates did not differ (HFNO 7.8% vs. LFNO 14.0%, *P* = 0.330), nor did SAEs (7.8% vs. 10.0%, *P* = 0.700). No serious adverse events were attributed to the oxygen delivery method. All outcomes see SDC, supplemental Table 1. BIS values were comparable, indicating similar sedation depth.

In this RCT, HFNO did not improve oxygenation compared with LFNO during PSA for AF catheter ablation. Hypoxaemia was rare, with a single event in the LFNO group; differences should therefore be interpreted cautiously and may reflect random variation rather than a true physiological effect.

Several methodological aspects may have influenced these findings. First, the study was terminated prematurely due to declining recruitment, resulting in a limited sample size and reduced statistical power. This limits the ability to draw definitive conclusions in this setting.

Second, airway management differences may also have influenced results. Airway adjuncts were allowed in LFNO but not during HFNO in order to avoid loss of pharyngeal pressure. During deep sedation upper airway collapse is common. The low positive airway pressure generated by HFNO, particularly with an open mouth, is unlikely to overcome upper airway obstruction in deeply sedated patients, potentially contributing to more HFNO-to-LFNO crossovers and impaired oxygen delivery. The difference in airway management may have masked potential benefits of HFNO observed in other procedural sedation settings. In addition, undiagnosed sleep-disordered breathing, which is highly prevalent in the atrial fibrillation population, may have had a greater impact in the HFNO group.^5^

Sedation depth may further complicate the comparison of oxygenation strategies. BIS monitoring demonstrated no differences between groups, suggesting comparable depth of sedation excluding the possibility of a systematic over-sedation in the HFNO group. Nonetheless, higher mean and maximum transcutaneous carbon dioxide levels were observed with HFNO, indicating greater hypoventilation.

Sedation-related complications were common and often mild and transient, consistent with prolonged deep procedural sedation. Our findings support the overall safety of PSA in this setting while emphasising the need for vigilant airway management regardless of oxygen delivery modality.

Current evidence on HFNO during PSA is mixed and dependent on the clinical setting^6,7,8^ (SDC, supplemental Table 2). Previous HFNO studies in endoscopy and bronchoscopy have shown less hypoxaemia, higher nadir SpO_2_, and fewer airway interventions compared with LFNO. However, these studies often involve lighter sedation levels than the deep sedation used in our cohort. Therefore, reported benefits of HFNO should be interpreted with caution and may not be generalisable to our setting.

In our limited investigation, we were unable to demonstrate a clear benefit of HFNO during AF ablation with PSA. Although HFNO appeared safe, it demonstrated no clinically meaningful advantage over LFNO. It is therefore concluded that HFNO does not overcome upper airway obstruction during deep sedation and should not be relied upon to maintain oxygenation during an airway collapse; appropriate airway management remains essential. Given the additional costs and environmental impact associated with HFNO, further studies addressing the limitations of our study are needed before firm conclusions regarding its routine use can be drawn. Future research should focus on selected patient groups and clinically relevant outcomes, including hypoxaemia, airway obstruction, procedural interruption, patient comfort, and cost-effectiveness, to determine which patients may derive meaningful benefit from HFNO.

## Supplementary Material

Supplemental Digital Content
