# Supplementary material for: High-flow versus low-flow nasal oxygen during procedural sedation for atrial fibrillation ablation: A randomised controlled trial
Source: Eur J Anaesthesiol Intensive Care. 2026 Jul 29;5(4):e0129. doi: 10.1097/EA9.0000000000000129 (PMC13568400; doi:10.1097/EA9.0000000000000129)
Supplement: Supplementary file 1 [file ejaic-5-e0129-s001.docx]

**Supplemental Table 1 Baseline SpO_2_**, **primary and secondary outcomes**

|  | *HFNO n=51* | | | *LFNO n=50* | | | | | *p-value* | |
| --- | --- | --- | --- | --- | --- | --- | --- | --- | --- | --- |
|  | *n* | % | Mean ± SD | *n* | % | | Mean ± SD | | Univariate |  |
| Baseline SpO2 % |  |  | 99.2% (±1.2) |  | |  | | 99.0 (±1.4) | 0.600 | |
| Primary outcome |  |  |  |  | |  | |  |  | |
| *Lowest SpO2 % (mean*±*SD)* |  |  | 89.6% (± 6.6) |  | |  | | 87.7% (±5.9) | 0.127 |  |
| *Duration lowest SpO2 seconds (mean*±*SD)* |  |  | 55.7 (± 99.4) |  | |  | | 41.3 (±54.2) | <0.001^a^ |  |
| *Duration lowest sp02 Quartile* |  |  |  |  | |  | |  |  | |
| *Q1-Q3* | 39 | 76.5 |  | 46 | | 93.9 | |  |  | |
| *Q4* | 12 | 23.5 |  | 3 | | 6.1 | |  | 0.023 |  |
| Secundary outcome |  |  |  |  | |  | |  |  | |
| *Cross over therapy* | 7 | 13.7 |  | 3 | | 6.0 | |  | 0.205 |  |
| *HypoxAemic events* | 0 | 0.0 |  | 1 | | 1.0 | |  | n.a. |  |
| *AUC incidence* | 0 | 0.0 |  | 1 | | 1.0 | |  | n.a. |  |
| *AUC (mean*±*SD)* |  | n.a. |  |  | | 240 | |  | n.a. |  |
| *Min TcCO_2_ (mean*±*SD)* |  |  | 4.9 (±0.7) |  | |  | | 4.9 (±0.8) | 0.824 |  |
| *Max TcCO_2_ (mean*± *SD)* |  |  | 7.8 (±1.0) |  | |  | | 7.4 (±0.9) | 0.017 |  |
| *Mean TcCO_2_ (mean*±*SD)* |  |  | 6.8 (±0.8) |  | |  | | 6.4 (±0.7) | 0.036 |  |
| DURATion PSA minutes *(mean*±*SD)* |  |  | 179.9 (±47.1) |  | |  | | 171.4 (±54.8) | 0.404 |  |
| DURATION PSA Quartile |  |  |  |  | |  | |  |  | |
| Q1-3 | 36 | 75.2 |  | 40 | | 80.0 | |  |  | |
| Q4 | 15 | 24.7 |  | 10 | | 20.0 | |  | 0.276 |  |
| Duration procedure minutes *(mean* ±*SD)* |  |  | 160.2 (±43.9) |  | |  | | 151.0 (±50.7) | 0.334 |  |
| DURATION PSA Quartile |  |  |  |  | |  | |  |  | |
| Q1-3 | 38 | 77.2 |  | 40 | | 80.0 | |  |  | |
| Q4 | 13 | 23.8 |  | 10 | | 20.0 | |  | 0.511 |  |
| ISAS *(mean*±*SD)* |  |  | 25.1 (±5.9) |  | |  | | 27.3 (±5.9) | 0.081^a^ |  |
| Patients comfort with oxYgen delivery device *(mean*±*SD)* |  |  | 4.00 [±0.8]^b^ |  | |  | | 4.00 [±0.8]^b^ | 0.396^c^ | |
| LIKERT scale 1 | 1 | 2.9 |  | 1 | | 3.3 | |  |  | |
| LIKERT scale 2 | n.a. | n.a. |  | 1 | | 3.3 | |  |  | |
| LIKERT scale 3 | 16 | 45.7 |  | 7 | | 23.3 | |  |  | |
| LIKERT scale 4 | 13 | 37.1 |  | 18 | | 60.0 | |  |  | |
| LIKERT scale 5 | 5 | 14.3 |  | 3 | | 10.0 | |  |  | |
| ACP user-friendliness oxygen delivery device *(mean*±*SD)* |  |  | 4.0 [±0.8]^b^ |  | |  | | 4.0 [±1.0]^b^ | 0.169^c^ | |
| LIKERT scale 1 | n.a | n.a |  | n.a | | n.a | |  |  | |
| LIKERT scale 2 | 6 | 19.4 |  | n.a | | n.a | |  |  | |
| LIKERT scale 3 | 1 | 3.2 |  | 4 | | 12.1 | |  |  | |
| LIKERT scale 4 | 19 | 61.3 |  | 21 | | 63.6 | |  |  | |
| LIKERT scale 5 | 5 | 16.1 |  | 8 | | 24.3 | |  |  | |
| ACP Difficulty maintaining oxygenation status *(mean*±*SD)* |  |  | 4.0 [±1.1]^b^ |  | |  | | 4.0 [±1.0]^b^ | 0.150^c^ | |
| LIKERT scale 1 | 1 | 3.2 |  | 1 | | 3.0 | |  |  | |
| LIKERT scale 2 | 3 | 9.7 |  | 3 | | 9.1 | |  |  | |
| LIKERT scale 3 | 5 | 16.1 |  | 8 | | 24.2 | |  |  | |
| LIKERT scale 4 | 10 | 32.3 |  | 16 | | 48.5 | |  |  | |
| LIKERT scale 5 | 12 | 38.7 |  | 5 | | 15.2 | |  |  | |
| EP satisfaction with catheter stabilisation *(mean*±*SD)* |  |  | 5.0 [±1.2]^b^ |  | |  | | 4.0 [±1.2]^b^ | 0.124^c^ | |
| LIKERT scale 1 | 1 | 4.0 |  | 1 | | 5.3 | |  |  | |
| LIKERT scale 2 | 1 | 4.0 |  | 2 | | 10.5 | |  |  | |
| LIKERT scale 3 | 2 | 8.0 |  | 3 | | 15.8 | |  |  | |
| LIKERT scale 4 | 6 | 24.0 |  | 6 | | 31.6 | |  |  | |
| LIKERT scale 5 | 15 | 60.0 |  | 7 | | 36.8 | |  |  | |

^a^Poisson regression; ^b^median [±IQR[; ^c^Mann–Whitney *U*; n.a.: not applicable; ISAS: Iowa Satisfaction with Anesthesia Scale
