# Supplementary material for: High-flow versus low-flow nasal oxygen during procedural sedation for atrial fibrillation ablation: A randomised controlled trial
Source: Eur J Anaesthesiol Intensive Care. 2026 Jul 29;5(4):e0129. doi: 10.1097/EA9.0000000000000129 (PMC13568400; doi:10.1097/EA9.0000000000000129)
Supplement: Supplementary file 2 [file ejaic-5-e0129-s002.docx]

**Supplemental Table 2** HFNO during PSA in various procedures

| **Study** | **Year** | **Setting** | **Sample Size** | **Comparator** | **Primary Outcome** | **Key Findings** |
| --- | --- | --- | --- | --- | --- | --- |
| **RCTs Cardiology** |  |  |  |  |  |  |
| Conway et al.,^1^ randomised controlled trial | 2022 | Cardiac device implantation with PSA | 126 | LFNO (5 l min^-1^, 100% O₂) | TcCO₂, hypoxaemia incidence, safety | HFNO safe; no significant improvement in oxygenation |
| Scheuermann *et al*.,^2^ randomised controlled trial | 2022 | Transcatheter aortic valve replacement (TAVR) with conscious sedation | 80 | Standard oxygen therapy | Hypoxaemia, hypercapnia, safety | HFNO did not significantly improve oxygenation or CO₂ clearance but was safe |
| **Systematic reviews / Meta-analysis** |  |  |  |  |  |  |
| Su *et al.*^3^, systematic review & meta-analysis | 2021 | Bronchoscopy with sedation | 257 (5 RCTs) | LFNO / conventional oxygen | Incidence of hypoxemic events, lowest SpO₂ | HFNO reduced hypoxaemia and improved nadir SpO₂. |
| Carron *et al.*,^4^ systematic review & meta-analysis | 2022 | Gastrointestinal endoscopy with deep sedation | 2,867 (6 RCTs) | LFNO / standard oxygen | Hypoxemic events, min SpO₂, airway interventions, procedure interruption | HFNO reduced hypoxaemia, airway interventions, and procedure interruptions. |
| Zhang *et al.*,^5^ systematic review & meta-analysis | 2022 | Digestive endoscopy with sedation | 2,998 (7 studies) | Standard nasal cannula (LFNO) | Hypoxemia incidence, airway interventions | HFNO lowered hypoxaemia rates and reduced need for interventions. |
| Tao *et al.,*^6^ systematic review & meta-analysis | 2022 | Endoscopic procedures with sedation | 4093 | LFNO / conventional oxygen | Oxygenation, desaturation, procedure interruption | HFNO improved oxygenation, reduced desaturation and interruptions. |
| Thiruvenkatarajan *et al.*^7^ systematic review & meta-analysis | 2023 | Procedural sedation (across specialties) | 4121 (15 RCTs) | Standard O₂ | Incidence of hypoxaemia, airway events | HFNO reduced risk of hypoxaemia across procedures but with moderate heterogeneity. |

1.Conway A, Collins P, Chang K, et al. High flow nasal oxygen during procedural sedation for cardiac implantable electronic device procedures: A randomised controlled trial. Eur J Anaesthesiol. 2021; 38:839-849.

2.Scheuermann S, Tan A, Govender P, et al. High-flow nasal oxygen vs. standard oxygen therapy for patients undergoing transcatheter aortic valve replacement with conscious sedation: a randomised controlled trial. Perioper Med (Lond). 2023; 12:11.

3.Su CL, Chiang LL, Tam KW, et al. High-flow nasal cannula for reducing hypoxemic events in patients undergoing bronchoscopy: A systematic review and meta-analysis of randomized trials. PLoS One. 2021; 16:e0260716.

4.Carron M, Tamburini E, Safaee Fakhr B, et al. High-flow nasal oxygenation during gastrointestinal endoscopy. Systematic review and meta-analysis. BJA Open. 2022; 4:100098.

5.Zhang YX, He XX, Chen YP, et al. The effectiveness of high-flow nasal cannula during sedated digestive endoscopy: a systematic review and meta-analysis. Eur J Med Res. 2022; 27:30.

6.Tao Y, Sun M, Miao M, et al. High flow nasal cannula for patients undergoing bronchoscopy and gastrointestinal endoscopy: A systematic review and meta-analysis. Front Surg. 2022; 9:949614.

7.Thiruvenkatarajan V, Sekhar V, Wong DT, et al. Effect of high-flow nasal oxygen on hypoxaemia during procedural sedation: a systematic review and meta-analysis. Anaesthesia. 2023; 78:81-92.
